# The Unitary Model for Estrogen Deficiency and the Pathogenesis of Osteoporosis: Is a Revision Needed?

**DOI:** 10.1002/jbmr.262

**Published:** 2010-10-06

**Authors:** Sundeep Khosla, L Joseph Melton, B Lawrence Riggs

**Affiliations:** Endocrine Research Unit, College of Medicine, Mayo ClinicRochester, MN, USA

**Keywords:** OSTEOPOROSIS, MENOPAUSE, AGING

## Abstract

Over a decade ago, we proposed a “unitary” model for the pathogenesis of osteoporosis that identified estrogen deficiency as the predominant cause of both the early, accelerated, and late slow phases of bone loss in postmenopausal women and as a contributing cause of the continuous phase of bone loss in aging men. While this was a plausible model then, new data over the intervening years suggest a need to modify these concepts. Indeed, based largely on rodent studies, a “revisionist” view of the pathogenesis of osteoporosis has been proposed recently that attempts a paradigm shift from the estrogen-centric model to one in which bone loss is largely independent of estrogen deficiency and is driven instead by cell-autonomous age-related factors. However, detailed clinical investigative studies using quantitative computed tomography demonstrate that the onset of cortical bone loss in humans is closely tied to estrogen deficiency; thus the estrogen-centric view is likely correct for cortical bone, which comprises over 80% of the skeleton and is the major structural determinant of fracture risk at most skeletal sites. By contrast, these same studies also demonstrate that trabecular bone loss begins in sex hormone–replete young adults of both sexes. This suggests that a significant proportion of trabecular bone loss is either estrogen-independent or, as suggested by some studies, requires higher levels for its regulation. In this perspective, we critically review these and other findings, leading us to conclude that our original model requires modification but not revision. © 2011 American Society for Bone and Mineral Research.

## Introduction

In 1998, we modified the original concept of type I versus type II osteoporosis proposed by Riggs and Melton in 1983(1) into a “unitary” model for the pathogenesis of involutional osteoporosis.(2) The key feature of this model was identification of estrogen deficiency as the cause of both the early, accelerated, and late slow phases of bone loss in postmenopausal women and as a contributing cause of the continuous phase of bone loss in aging men. The accelerated phase in women was believed to be most apparent during the first 3 to 5 years after menopause, involved disproportionate loss of trabecular bone, and was attributed mainly to loss of the direct restraining effects of estrogen on bone cell function. In this model, the ensuing slow phase continued throughout life in women, involved proportionate losses of trabecular and cortical bone, and was postulated to be caused by progressive secondary hyperparathyroidism induced by loss of estrogen action on extraskeletal calcium homeostasis that resulted in net calcium wasting and increases in dietary calcium intake required to maintain bone balance. Given evolving understanding of the role of estrogen regulation of bone metabolism in men and the demonstration that their serum bioavailable (non–sex hormone–binding globulin–bound) estrogen levels decline with aging, estrogen deficiency also was believed to contribute substantially to the continuous bone loss of aging men. In both genders, estrogen deficiency increased bone resorption and also impaired compensatory increases in bone formation.

While this was a plausible model based on the data available at the time, the past 12 years have witnessed significant advances in our understanding of both the pattern and underlying mechanisms of bone loss with aging. At the clinical investigative level, advances in imaging techniques have allowed for a more rigorous examination of the distinct patterns of cortical versus trabecular bone loss at various stages of life, which was not possible using 2D dual-energy X-ray absorptiometry (DXA). At a mechanistic level, there has been a significant expansion of our knowledge regarding the fundamental mechanisms of estrogen action on bone and the potential interactions of estrogen deficiency with underlying age-related changes in bone. Indeed, these advances in our knowledge of aging mechanisms have prompted Manolagas to propose a “revised” model of the pathogenesis of osteoporosis.(3) This new model attempts a paradigm shift from the estrogen-centric account of the pathogenesis of involutional osteoporosis to one in which age-related mechanisms intrinsic to bone (specifically, oxidative stress) are the major driving force for bone loss throughout life, albeit aggravated by effects of sex steroid deficiency. Here we reexamine our original unitary model in light of the intervening clinical and basic mechanistic data and, where appropriate, provide modifications to it.

## Patterns of Age-Related Bone Loss from New Imaging Approaches

**Fig. 1:**
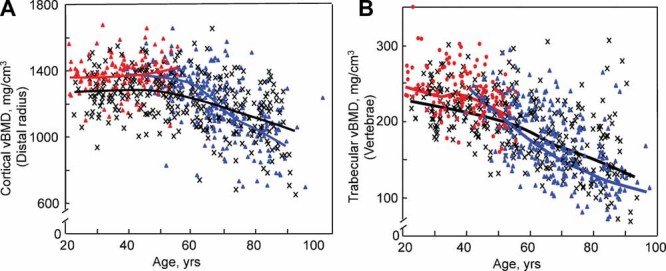
(*A*) Values for cortical vBMD at the distal radius in a population sample of Rochester, MN, women and men between the ages of 20 and 97 years. Individual values and smoother lines are given for premenopausal women in red, for postemenopausal women in blue, and for men in black. (*B*) Values for vertebral trabecular vBMD in the same cohort. Color code is as in panel *A*. All changes with age were significant (*p* < .05). (Reproduced from Riggs et al.(4))

Although extremely useful clinically, DXA cannot distinguish cortical from trabecular bone, thereby limiting its utility in assessing independent changes in these compartments. Thus, in a population-based study, we assessed age- and sex-specific changes in bone structure by quantitative computed tomography (QCT) at the distal radius, distal tibia, lumbar spine, and femoral neck.(4) Key findings from this study relevant to the issue of estrogen deficiency versus aging effects on cortical and trabecular bone changes over life are shown in Figure 1. Figure 1*A* shows cortical bone changes at the distal radius as an example (similar changes were noted for cortical bone at the distal tibia and femoral neck). In this cross-sectional analysis, cortical bone remained stable in both women and men until midlife. Thereafter, associated with the menopause in women and, presumably at least in part, with age-related changes in sex steroid levels in men (discussed in detail in a subsequent section), there were progressive decreases in cortical volumetric bone mineral density (vBMD) in both sexes. Figure 1*B* shows comparable changes in trabecular vBMD at the spine (with similar changes noted in trabecular bone at other sites, including the femoral neck, distal radius, and distal tibia). In marked contrast to changes in cortical bone, trabecular bone loss began in young adulthood in both sexes, at a time when sex steroid levels are, by definition, normal.

**Fig. 2:**
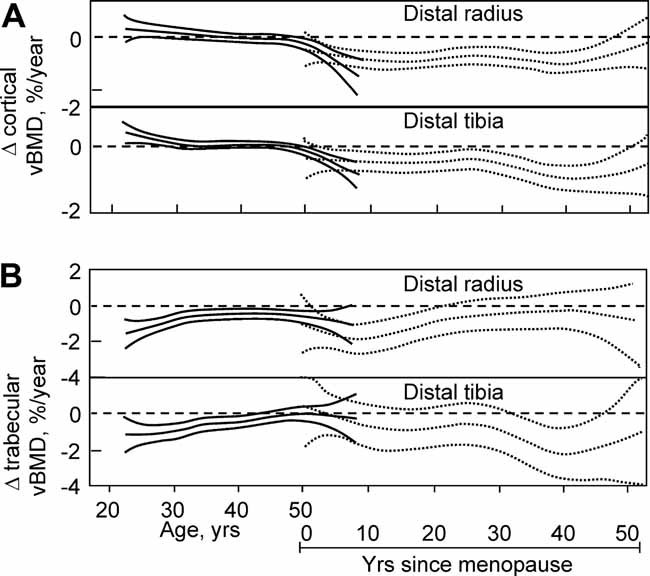
(*A*) Age-specific changes in cortical vBMD at the distal radius and tibia in women. Data are shown with a smooting spline and the 95% CI. Premenopausal women (*solid lines*) are plotted against age in years, whereas postmenopausal women (*broken lines*) are plotted against years since menopause. (*B*) Corresponding changes in trabecular vBMD at the distal radius and distal tibia. (Adapted from Riggs et al.(5))

We subsequently validated these cross-sectional findings in a longitudinal analysis of rates of bone loss at multiple skeletal sites using QCT.(5) In this study, vBMD of cortical and trabecular bone at the distal radius and tibia was measured annually over 3 years and at the lumbar spine at baseline and 3 years. We summarize here the findings in the women, although there was a similar concordance between the cross-sectional and longitudinal findings in the men.(5) Figure 2*A* shows annualized rates of change in cortical vBMD at the distal radius and tibia in women. In postmenopausal women, the data are plotted as a function of years after menopause, although the pattern was very similar when plotted as a function of age. Consistent with the cross-sectional findings, in premenopausal women there was minimal cortical bone loss until the perimenopausal interval, as indicated by the inclusion of zero in the 95% confidence interval (CI) over that range. Statistically significant bone loss occurred thereafter, with a relatively constant subsequent rate of loss. Figure 2*B* shows the corresponding changes for trabecular vBMD with age and menopausal status. Again consistent with the cross-sectional findings, trabecular bone loss assessed longitudinally was evident in young women (in the third decade), with an apparent acceleration during the perimenopausal interval. Of interest, at least at the distal radius and tibia, the rate of trabecular bone loss was maximal in the third decade and declined until midlife, when it again accelerated. In elderly women, trabecular bone loss waned, possibly owing to exhaustion of trabecular bone at these appendicular sites. Longitudinal changes in trabecular bone at the spine were qualitatively similar, although rates of bone loss at this central site did not decrease with advancing age.(5)

These cross-sectional and longitudinal findings have important implications for the contention by Manolagas that total bone loss over life is largely independent of sex steroids.(3) This does appear to be the case for trabecular bone because our cross-sectional and longitudinal data clearly indicate that trabecular bone loss begins in young-adult life, in the setting of normal sex steroid levels.(4,5) However, this is not the case for cortical bone because the onset of cortical bone loss is clearly associated with menopause in women.(4,5) This is not to imply that additional sex steroid–independent factors (discussed in detail below) do not compound the effects of sex steroid deficiency and further accelerate cortical bone loss following the onset of estrogen deficiency. However, the fundamental view of bone loss we proposed over a decade ago that assigns a central role to estrogen deficiency does appear to be correct for cortical, if not for trabecular, bone. Furthermore, since cortical bone comprises over 80% of the adult skeleton(6) and is increasingly recognized as a major structural determinant of bone strength,(7,8) it is perhaps not surprising that fracture patterns over life closely parallel the observed changes in cortical bone. Specifically, numerous studies have consistently found that the incidence of all fractures, including vertebral fractures (where the “cortical shell” may contribute significantly to bone strength(7)), remains relatively low until menopause in women, at which time incidence rates increase dramatically and continue to climb through remaining life.(9,10) Moreover, the marked perimenopausal increases in fracture incidence are not due to bone loss alone but also result from the destabilizing effect on bone strength of microarchitectural disruption induced by increases in bone turnover consequent to estrogen deficiency.(11)

## Treatment Studies in Humans and Mice

While the observational data described above are consistent with the estrogen-centric model (at least for cortical bone), it is difficult to test this hypothesis in interventional studies in humans given the long duration needed to dissect effects of estrogen deficiency from those of aging per se. Nonetheless, Richelson and colleagues(12) performed a unique case-control analysis over 25 years ago to address this issue. They studied 14 women who had undergone oophorectomy during young adulthood, 14 normal perimenopausal women, and 14 normal postmenopausal women (mean ages 54, 52, and 73 years, respectively; mean duration of estrogen deficiency 22, 0.3, and 22 years, respectively). In this design, the oophorectomized and perimenopausal women were matched for age but differed in the duration of estrogen deficiency by over 20 years. By contrast, the oophorectomized and postmenopausal women were matched for duration of estrogen deficiency but differed in age by almost 20 years. The key finding of this study was that areal BMD (aBMD) by DXA at multiple sites (ie, midradius, femoral neck, and lumbar spine) was virtually identical in the oophorectomized and postmenopausal women (despite their difference in age but concordance in years since menopause), and aBMD in both groups was markedly lower than in the perimenopausal women. These data thus indicated that duration of estrogen deficiency, rather than age, was the critical determinant of bone loss, at least in the first two decades after menopause. Since this study used DXA for aBMD measurements, however, it could not evaluate the role of estrogen deficiency versus aging separately on loss of trabecular versus cortical bone.

We recently extended the observational and limited interventional human data to a mouse model to address this issue more directly.(13) While female mice do not have the equivalent of menopause, they do undergo reproductive senescence, becoming essentially acyclic by 11 to 16 months of age.(14,15) This is accompanied by significant reductions in circulating estradiol levels, although in contrast to humans(16) the decreases in circulating estradiol levels in aging female mice are not as large as in women and thus are more difficult to detect. For example, Nelson and colleagues(17) found that while serum estradiol levels in aging (10- to 12.5-month-old) C57BL/6J mice on day 1 (proestrus) and day 2 (estrus) of the estrus cycle were similar to those found in younger mice (ages 5.5 to 7.5 months), estradiol levels on day 3 and the preovulatory estradiol rise beginning on day 4 were reduced in older mice by 80% and 45%, respectively. Thus female aging mice do in fact develop estrogen deficiency, although this is clearly not as profound as that observed in postmenopausal women. Interestingly, age-related declines in vertebral and femoral trabecular bone begin in female mice by 2 months of age,(18) at a time of sex steroid sufficiency, again suggesting that trabecular bone loss in mice, as in humans, may be relatively independent of prevailing estrogen levels.

**Fig. 3:**
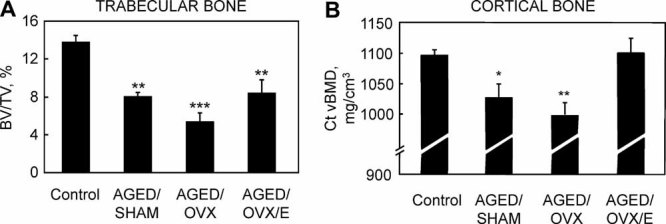
(*A*) Trabecular bone volume/total volume (BV/TV) and (*B*) cortical vBMD (Ct vBMD) in 6-month-old (control) mice, aged/sham-operated mice, aged/ovariectomized (ovx) mice, and aged/ovariectomized mice replaced throughout life with physiologic doses of estradiol using continuous-release pellets (aged/ovx/E). *p < .05; **p < .01; and ***p < .001 versus young controls. (Adapted from Syed et al.(13))

To dissect estrogen versus aging effects on bone loss in mice, we sham-operated, ovariectomized, or ovariectomized and estrogen-replaced (with physiologic doses of estradiol using continuous-release pellets) female C57/BL6 mice at 6 months of age and followed them to age 18 to 22 months (the stage of murine senescence). Six-month-old intact control mice were euthanized to define baseline parameters. Figure 3*A* shows the structural trabecular bone data at the spine analyzed by micro–computed tomography (µCT). As compared with the young control mice, trabecular bone volume/total volume (BV/TV) was 42% lower in the aged/sham mice and 61% lower in the aged/ovariectomized mice, confirming previous findings of significant trabecular bone loss over life in mice(18) and demonstrating acceleration of this loss with profound estrogen deficiency following ovariectomy, as is the case in humans.(16) Interestingly, however, BV/TV was identical in the aged/ovariectomized/estrogen group to that in the aged/sham-operated group and significantly lower than in the young control mice. These data thus clearly demonstrated that age-related trabecular bone loss at the spine is unaltered by maintaining constant estrogen levels over life. Likewise, cortical vBMD at the tibial diaphysis was significantly lower in the aged/sham-operated and aged/ovariectomized mice compared with the young control mice (Fig. 3*B*). In contrast to the spine trabecular bone findings, however, the aged/ovariectomized/estrogen mice had cortical bone parameters identical to those in the young control mice and significantly greater than those in the aged/sham-operated (or aged/ovariectomized) mice, thus demonstrating that maintaining constant estrogen levels over life can prevent “age-related” cortical bone loss in mice. These direct interventional data in mice are remarkably consistent with our observational data in humans described earlier. Thus, in both species, trabecular bone loss over life is largely independent of prevailing levels of endogenous estrogen (although it is accelerated by estrogen deficiency), whereas cortical bone loss appears to be principally related to estrogen deficiency.

## Studies in Men

In our original model, we proposed that declining bioavailable estrogen levels also made a substantial contribution to age-related bone loss in men.(2) Considerable evidence since then provides strong support for this postulate, and interestingly, studies in men may provide insights into why trabecular and cortical bone may respond differently to estrogen and estrogen deficiency. Thus a number of observational studies now have demonstrated that serum estrogen levels are important determinants of bone mass(19–26) and bone loss(27–29) in men. Moreover, short-term interventional studies in which men are made hypogonadal and are selectively replaced either with estrogen or testosterone have demonstrated the importance of estrogen in regulating both bone formation and bone resorption in adult men.(30,31)

**Fig. 4:**
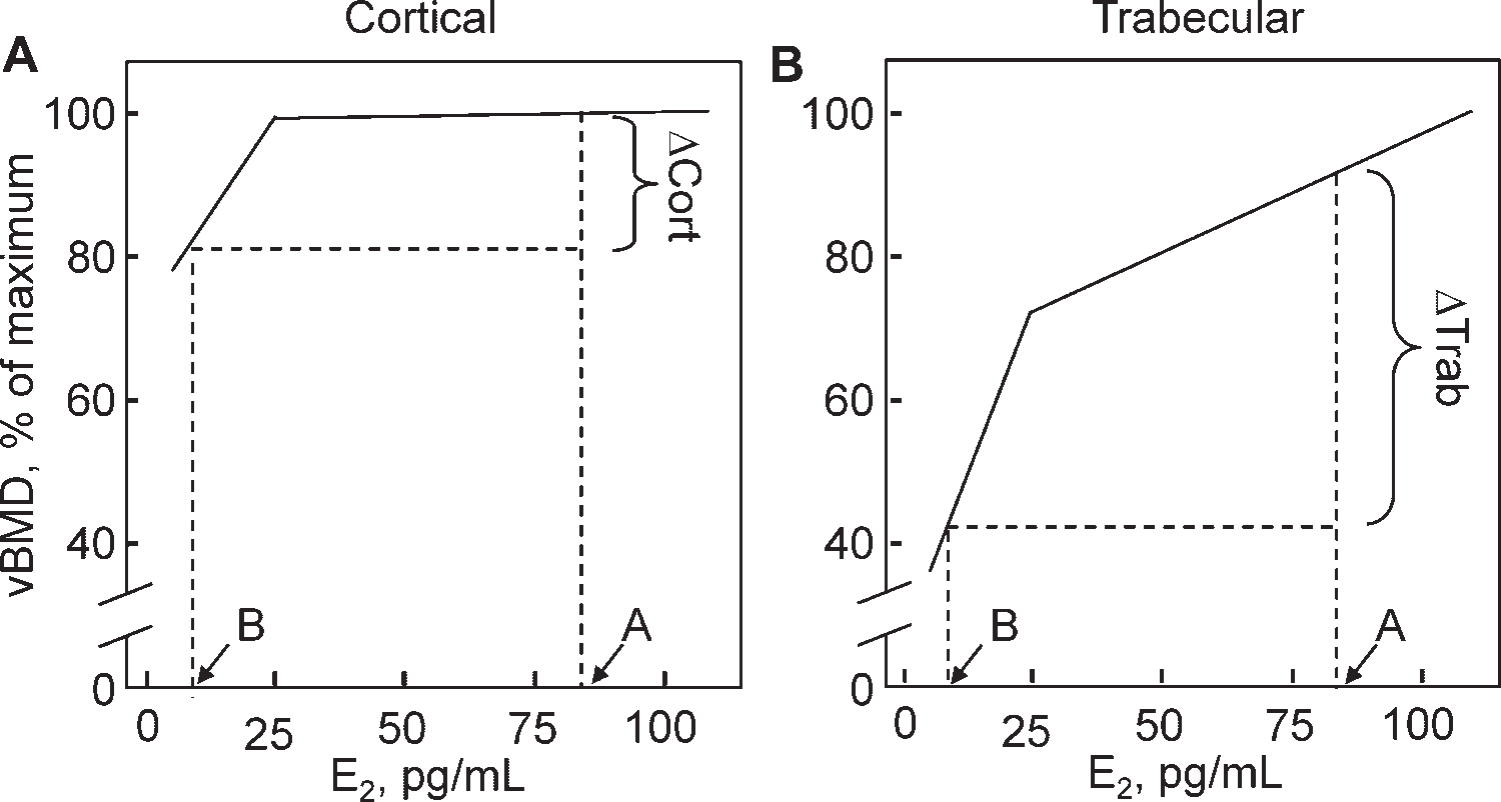
Schematic illustration, based on the data of Khosla and colleagues,(36) of the relationship between (*A*) cortical vBMD and (*B*) trabecular vBMD and serum estradiol levels. Note that while cortical vBMD is correlated with estradiol levels at low estradiol levels, no relationship is evident at high estradiol levels, consistent with a threshold below which cortical bone becomes estrogen-deficient. By contrast, trabecular vBMD remains correlated with estradiol levels at low and high serum estradiol levels, suggesting either the absence of a threshold or a threshold considerably higher than that present for cortical bone. Shown also are the relative changes in cortical (▵cort) and trabecular (▵trab) bone as estrogen levels fall from estrogen sufficiency (point *A*) to estrogen deficiency (point *B*).

There is also increasing evidence for a threshold for estrogen effects on bone in normal adult men. Indeed, it has been easier to tease out dose-response relationships between serum estradiol levels and bone turnover or bone mass in men, who span a range of estradiol levels that includes this apparent threshold; by contrast, premenopausal women are well above this threshold, whereas postmenopausal women are considerably below it, making it difficult to define the overall relationship between serum estradiol levels and bone metabolism.(27) Thus studies using the selective estrogen receptor modulator (SERM) raloxifene have found that men with low endogenous estradiol levels (less than approximately 25 pg/mL) tend to have a decrease in bone-resorption markers, but men with endogenous estradiol levels above this value have the opposite response following raloxifene treatment—namely, an increase in bone resorption.(32,33) Moreover, rates of bone loss(27,28) and fracture risk(34,35) seem to be highest in men with estradiol levels below approximately 20 to 25 pg/mL using immunoassays (or approximately 16 pg/mL using mass spectroscopy(35)), and variations in estradiol levels above this range do not appear to be related to bone loss or fracture risk in aging men. However, studies using QCT also have provided evidence for differences in the dose relationships between serum estradiol levels and trabecular verus cortical bone.(36) There appears to be a clear threshold for this relationship in cortical, but not in trabecular, bone, or at least the relationship between serum estradiol and cortical bone plateaus at much lower estradiol levels (Fig. 4*A*) than the relationship between serum estradiol levels and trabecular bone (Fig. 4*B*). Thus one possible explanation for ongoing trabecular bone loss throughout life in women and men may be that trabecular bone is intrinsically less sensitive to estrogen than cortical bone in terms of all the pleiotropic effects of estrogen in maintaining bone mass. As such, trabecular bone loss occurs even in the setting of “normal” estrogen levels in young-adult life, whereas cortical bone is maintained until the onset of estrogen deficiency following menopause or age-related decreases in bioavailable estrogen levels late in life in men.(37)

## Mechanisms of Action of Estrogen on Bone Cells

To understand how the differential effects of estrogen on cortical and trabecular bone might occur and how estrogen might interact with other age-related processes, it is important to briefly review what we currently understand regarding estrogen regulation of bone remodeling. Estrogen has three fundamental effects on bone metabolism: (1) It inhibits the activation of bone remodeling and the initiation of new basic multicellular units (BMUs); (2) it inhibits differentiation and promotes apoptosis of osteoclasts, thereby reducing bone resorption; and (3) while estrogen suppresses self-renewal of early mesenchymal progenitors, it promotes the commitment and differentiation and prevents apoptosis of osteoblastic cells, thereby maintaining bone formation at the cellular level. Each of these actions of estrogen is reviewed briefly below.

At the tissue level, estrogen clearly reduces bone turnover, both histologically and as reflected by changes in bone turnover markers.(38) Given increasing evidence that osteocytes may regulate the activation of bone remodeling via connections with bone lining cells,(39) it is likely that the antiremodeling effects of estrogen are mediated via the osteocyte. Indeed, withdrawal of estrogen is associated with increased apoptosis of osteocytes both in rodents(40) and in humans,(41) although the specific molecular mechanisms by which this then leads to increased remodeling on the bone surface remain unclear. Of interest, recent studies have shown that serum estradiol levels are inversely associated with serum levels of the key inhibitor of Wnt signaling produced by osteocytes, sclerostin,(42) and estrogen treatment of postmenopausal women reduces circulating sclerostin levels.(43) Moreover, there is growing evidence that Wnt/β-catenin signaling is important for the ability of the osteocyte to respond to mechanical strain, and this response also depends on estrogen receptor α (ERα).(44) Thus there is likely important cross-talk between estrogen and Wnt signaling pathways mediated by the osteocyte that warrants further study.

In addition to inhibiting the activation of bone remodeling, estrogen also directly and indirectly suppresses bone resorption. Thus, by increasing osteoprotegerin (OPG)(45) and decreasing receptor activator of nuclear κB ligand (RANKL)(46) production by osteoblastic cells, as well as by suppressive effects on the production of tumor necrosis factor α (TNF-α)(47) and other proresorptive cytokines,(48) estrogen reduces osteoclastogenesis, at least in part, via effects on osteoblasts and perhaps also on T cells.(49) In addition, estrogen also modulates RANK signaling in osteoclastic cells(50,51) and induces apoptosis of osteoclasts,(52,53) thereby having direct effects on osteoclastic cells.

Finally, estrogen is clearly important for the maintenance of bone formation. Perhaps the most direct evidence for this comes from human studies showing that acute (3 weeks) estrogen deficiency either in women(54) or in men(30) is associated with a fall in bone-formation markers. However, owing to the subsequent “coupling” of bone formation with resorption, bone formation increases over time so that when chronically estrogen deficient women are studied, both bone-resorption and bone-formation markers are increased.(55) It also appears that the effects of estrogen on progenitor and osteoblastic cells may be stage-specific. Thus, consistent with the overall effects of estrogen on reducing bone remodeling, estrogen reduces the self-renewal of early mesenchymal progenitors.(56) Perhaps the most consistent effects of estrogen, however, are on inducing commitment of precursor cells to the osteoblast at the expense of the adipocyte lineage(57,58) and on preventing apoptosis of osteoblastic cells.(40) Estrogen also has been shown to enhance osteoblast differentiation,(58) although data on the effects of estrogen on osteoblastic differentiation are more variable and seem to depend on the model system used.

## Cellular Basis for Differential Effects of Estrogen on Trabecular versus Cortical Bone

Although trabecular bone appears to be relatively independent of regulation by estrogen levels present in young-adult women, it contains estrogen receptors, and its loss is accelerated, at least transiently, by menopause. This raises the possibility that trabecular bone is partly regulated by estrogen but with a higher threshold level than for cortical bone. The challenge is to try to explain why trabecular and cortical bone might have different sensitivities to estrogen. One explanation may come from studies by Bord and colleagues(59) showing that whereas osteocytes and osteoblasts in developing human cortical bone predominantly expressed ERα, the same cells in trabecular bone expressed not only ERα but also significantly higher levels of ERβ than were present in cortical bone. While ERβ itself may not directly regulate bone turnover,(60) its main role may be to modulate the action of ERα because ERα/β heterodimers appear to be less sensitive to estrogen than ERα homodimers.(61) Thus, if bone cells (ie, osteocytes, osteoblasts, and osteoclasts) in trabecular bone express more ERβ than these cells in cortical bone, trabecular bone cells would be less sensitive to all the estrogen actions on bone noted earlier. Consequently, higher estrogen levels would be needed to preserve trabecular as compared with cortical bone, which is entirely consistent with the dose relationship depicted in Fig. 4. Indeed, Windahl and colleagues(62) found that while BV/TV of trabecular bone at the femur decreased by 51% between the ages of 11 weeks and 12 months in wild-type mice, female *ERβ* knockout mice were partially protected against age-related trabecular bone loss and lost only 28% of BV/TV at this site. These findings in mice are, in fact, entirely consistent with earlier data in women using QCT, which found that a fourfold higher dose of estrogen was required to prevent trabecular as compared with cortical bone loss following oophorectomy.(63)

From an evolutionary perspective, why might natural selection have derived an estrogen-regulated process that leads to the greater proportional availability of calcium reserves in trabecular as compared with cortical bone? If bone loss must occur to meet the requirements of calcium homeostasis, cortical bone may be relatively protected because it carries a greater skeletal load and supports locomotion. Consequently, its loss likely would have a greater impact on species survival. Trabecular bone thus would be the preferred source of the additional calcium required to maintain homeostasis when this cannot be accomplished by increased intestinal absorption or decreased urinary excretion. This is particularly important when the calcium deficit is more extreme. For example, in lactation, serum estrogen levels fall and bone is lost to supply the large requirement for calcium in breast milk.(64,65) As estrogen levels decline, the dose relationship in Fig. 4 would predict that relatively more bone would be lost (and bone calcium mobilized for breast milk) from trabecular bone while preserving cortical bone. This is exactly what is observed in lactating women.(64)

Another situation that results in a major stress to calcium homeostasis is pregnancy, where large amounts of calcium are required for growth of the fetal skeleton. aBMD measurements obtained before and after pregnancy show that there is differential loss from predominantly trabecular bones of the axial skeleton, whereas there was no loss of predominantly cortical bone in the appendicular skeleton.(66) The homologue of the physiologic processes that provide relative protection for cortical bone as compared with trabecular bone during mammalian reproduction may have evolved in the oviparous ancestors of mammals. The egg-laying cycle of protomammals in the Triassic Era probably was similar to that which occurs in modern birds. In the weeks prior to egg laying in the hen, a large amount of endosteal trabecular bone is formed, which then is rapidly resorbed to provide calcium for eggshell mineralization prior to egg laying.(67) Thus the observed differential regulation of trabecular and cortical bone by estrogen makes eminent sense from an evolutionary perspective and from the role played by bone as a storage site for calcium needed for homeostasis.

## Intrinsic Aging Changes in Bone

As noted earlier, the revisionist model of Manolagas highlights the importance of cell-autonomous changes in bone that contribute to bone loss with aging but are largely independent of the sex steroid levels that are present in young-adult women and men.(3) We fully agree that such changes are important mediators of age-related bone loss, although they likely interact with the effects of estrogen deficiency. In a series of studies, Almeida and colleagues(40) have shown that in mice, aging is associated with an increase in markers of oxidative stress in osteoblastic cells, which results in an increase in Forkhead box O (FoxO) transcription factors. While FoxO induction is critical in the defense against oxidative stress, these investigators also found that by competing for cellular β-catenin, the increase in FoxO expression leads to a reduction in Wnt signaling in bone. Given the key role of Wnts in bone metabolism,(68) these findings suggest that oxidative stress, which increases with aging and is accentuated by sex steroid deficiency,(40) may be an important factor leading to impaired bone formation with aging.

There also has been considerable interest recently in the role of the nutrient-sensing nicotine adenine dinucleotide (NAD)–dependent protein deacetylases sirtuins in aging phenotypes in a number of tissues.(69) Thus it is of interest that Edwards and colleagues(70) have found that mice with global deletion of sirtuin 1 (*Sirt1*) have a decrease in bone mass associated with decreased bone formation and increased bone resorption. In further studies, these investigators showed that osteoblast-specific deletion of *Sirt1* results in low bone formation, whereas deletion of *Sirt1* in osteoclast precursor cells leads to an increase in bone resorption.(71) These findings therefore demonstrate that age-related changes in Sirt-1 activity also may contribute to age-related bone loss, at least in rodent models. Additional support for this hypothesis comes from data showing that the Sirt-1 agonist resveratrol results in preservation of BMD in aging mice(72) and can prevent ovariectomy-induced bone loss.(73)

There are, however, two caveats on these studies. First, the studies of Almeida and colleagues(40) were done in mice whose bone phenotype with aging appears to differ from that of aging humans in an important aspect. Although the aging C57/BL6 mice used by Almeida and colleagues(40) had increases in oxidative stress, bone remodeling was decreased in these female mice rather than increased, as occurs in aging women.(20)

The second caveat concerns the interaction of aging-dependent senescent changes with estrogen deficiency. As shown by many investigators, age-dependent oxidative stress increases cell senescence, and one of the major actions of estrogen is to decrease the formation of reactive oxygen species both in bone cells(40,74) and in a variety of other estrogen-responsive tissues. Thus it may be difficult to determine the relative contributions of aging-dependent increases of reactive oxygen species and those which are caused or enhanced by estrogen deficiency. Although only limited data exist on the role of estrogen deficiency in humans older than age 70 years, these do suggest that estrogen deficiency continues to play a major role in high levels of bone turnover, decreased BMD, and increased fracture risk in both sexes. The increases in bone turnover with aging in women and men correlate inversely with levels of serum estrogen. In large epidemiologic studies in elderly women(75) and men,(35) those with the lowest estrogen levels had the lowest bone density and highest risk of fractures. To address the issue of the relative effects of aging and estrogen deficiency on age-related increases in bone turnover, McKane and colleagues(76) studied three groups of 30 women: a premenopausal group (age 32 years), an untreated postmenopausal group, and an estrogen-treated postmenopausal group; the two postmenopausal groups had an average age of 75 years. Bone-resorption markers were similar in the premenopausal and estrogen-treated women but were significantly increased in the untreated group. These findings again suggest that estrogen deficiency (rather than aging per se) is more important for age-related increases in bone resorption.

While much more work is needed, it is nonetheless clear from these studies that there are underlying processes of skeletal aging that likely occur independent of the effects of estrogen. Additional studies examining other aspects of organismal aging, such as the accumulation in various tissues of senescent cells that produce a number of proinflammatory cytokines(77) and the effects of these cells and their secreted products on skeletal aging, need to be done.

## Role of Secondary Hyperparathyroidism

In our original model,(2) we postulated that the late phase of bone loss in aging individuals was mediated by the known increases in serum parathyroid hormone (PTH) levels in women and in men. Evidence in support of this came from studies demonstrating that suppression of PTH secretion by intravenous calcium infusion abolished the differences in bone-resorption markers between young and elderly women,(78) strongly suggesting that at least the increase in bone resorption (if not the impairment in bone formation) in aging women was PTH-dependent. We further suggested that the age-related increase in PTH secretion was due, in turn, to the loss of the effects of estrogen on extraskeletal calcium homeostasis. Specifically, estrogen was known to enhance intestinal(79) and renal(80) calcium absorption; conversely, chronic estrogen deficiency would be expected to lead to negative calcium balance and increases in PTH levels. A further critical test of this concept came from studies from our own group in which 42 elderly women (mean age 69 years, approximately 21 years postmenopause) were randomly assigned to groups receiving the potent aromatase inhibitor letrozole or placebo for 6 months.(81) Letrozole treatment reduces serum estrone and estradiol levels to near-undetectable levels. In response to this, compared with the placebo group, the bone-resorption marker urinary deoxypyridinoline increased by 22% (*p* = .002), demonstrating the importance of even the low residual estrogen levels present in late postmenopausal women in regulating bone resorption. Of importance, however, if the major effects of the residual estrogen in these women were on extraskeletal calcium homeostasis, further reducing these estrogen levels would be predicted to increase serum PTH levels owing to worsening calcium balance from decreased intestinal and renal calcium absorption. In fact, we observed a 22% reduction in serum PTH levels in the letrozole-treated women compared with the placebo-treated women owing to loss of the direct restraining effects of estrogen on bone resorption and the subsequent flux of calcium out of bone. Conversely, Lufkin and colleagues(38) reported that treatment of postmenopausal osteoporotic women with transdermal estrogen resulted in a trend toward increased PTH values. While secondary hyperparathyroidism with aging may contribute importantly to age-related bone loss,(78) these findings thus demonstrated that the direct skeletal actions of estrogen may be more dominant even in elderly women. It is also important to note that vitamin D deficiency with aging contributes independently to secondary hyperparathyroidism.(82) In addition, serum androgen levels in men are associated with serum 25-hydroxyvitamin D levels, and both hormones have a concordant seasonal variation,(83) suggesting important links between the vitamin D and sex steroid axes that warrant further investigation.

## Additional Factors Contributing to Perimenopausal Bone Loss

While menopausal bone loss in this Perspective has been considered largely in the context of estrogen deficiency, we do recognize that there are a number of additional hormonal changes during menopause that may contribute to bone loss. Thus the observation that early menopausal bone loss begins even when serum estradiol levels are normal led to the hypothesis a number of years ago by Prior and colleagues(84) that luteal phase defects and reductions in progesterone levels during perimenopause contribute to bone loss during this period. In addition to progesterone, androgen levels also decrease during the menopausal transition(85) and could contribute to bone loss. Recently, Perrien and colleagues(86) demonstrated marked reductions in serum inhibins (A and B) during menopause in women and found that the decreases in inhibin levels were associated with increases in bone turnover markers. In addition, Ebeling and colleagues(87) have found that increases in bone-resorption markers in perimenopausal women were best correlated not with serum estradiol levels but rather with follicle-stimulating hormone (FSH) levels. Subsequent data from the Study of Women's Health Across the Nation (SWAN) showed that spine and hip aBMD losses during the menopausal transition were most strongly related to the interaction between initial FSH levels and longitudinal FSH changes and not to estradiol or androgen levels.(88) These findings raised the possibility that FSH may have direct effects on bone; however, as noted by the authors of that study,(88) FSH also could be a better predictor of aBMD changes during perimenopause than estradiol because it may serve as a more robust proxy measure of ovarian dynamics involving estradiol than single estradiol measurements. In rodent studies, Sun and colleagues examined the skeletal phenotype of FSH receptor null (FORKO) mice and found that despite being hypogonadal, these mice had normal bone mass.(89) These investigators also found that osteoclasts and their precursors possessed FSH receptors and that FSH [but not luteinizing hormone (LH)] increased osteoclast formation and function in vitro, and they concluded that high circulating FSH levels caused hypogonadal bone loss.(89) By contrast, Gao and colleagues(90) subsequently found that the FORKO mice did have reduced bone mass; moreover, bilateral ovariectomy reduced the elevated circulating testosterone levels in the FORKO mice and decreased bone mass to levels indistinguishable from those in ovariectomized control mice. These investigators came to the opposite conclusion from that of Sun and colleagues,(89) namely, that sex steroids regulated bone turnover in the FORKO mice independently of any bone-resorptive action of FSH. Consistent with this, Drake and colleagues(91) found that suppression of FSH levels in postmenopausal women into the premenopausal range for 4 months failed to reduce bone-resorption markers, indicating that FSH is likely not an important regulator of bone resorption in humans. While FSH is unlikely to contribute to postmenopausal bone loss, the precise roles of changes in progesterone, androgen, and inhibin levels in enhancing the effects of estrogen deficiency on bone loss during the perimenopausal period remain to be fully defined.

## Summary and Conclusions

In this Perspective we have sought to update our original unitary model for the pathogenesis of osteoporosis(2) in light of the new evidence that has emerged over the past decade. We would submit that the revisionist view proposed by Manolagas,(3) which states that age-related bone loss is mostly independent of estrogen deficiency and is driven instead by cell-autonomous age-related factors may be largely correct for trabecular bone. Indeed, we had recognized previously that because trabecular bone loss preceded the onset of sex steroid deficiency in both sexes, it may be due to estrogen-independent processes(5) or, as suggested by other studies, trabecular bone may require higher levels for its regulation. However, for cortical bone, which comprises over 80% of the skeleton(6) and is likely the major contributor to overall fracture risk, the collective data still support the view that estrogen deficiency is the major cause of postmenopausal bone loss in women and age-related bone loss in both sexes. We fully agree, however, that intrinsic, cell-autonomous aging processes in bone, as in other tissues, likely contribute and accentuate aging-dependent bone loss in both trabecular and cortical bone. Additionally, as we recognized before,(2,16) a host of secondary factors (eg, vitamin D deficiency and other secondary causes of osteoporosis) also have an impact on the underlying bone loss with aging in each individual. Finally, while secondary hyperparathyroidism undoubtedly contributes to age-related increases in bone resorption, the evidence does point to ongoing direct effects of even the low residual estrogen levels present in postmenopausal women in restraining bone turnover and continuing to play an important role in regulating bone metabolism.

In summary, in light of new clinical investigative and basic mechanistic data, we have appropriately modified our original model. Our modified unitary model, which continues to emphasize the central role of estrogen, and the revisionist model,(3) which emphasizes aging-dependent changes in bone cells in the pathogenesis of osteoporosis are likely complementary—and each is more appropriate in specific skeletal compartments. Moreover, estrogen deficiency and underlying aging processes in bone likely interact to accentuate the deleterious effects of each. The challenge remains to better understand the fundamental mechanisms by which these processes cause bone loss individually and in combination and, based on this understanding, to continue to develop more effective preventive and treatment approaches.
